# Effect of Core–Shell Ceria/Poly(vinylpyrrolidone) (PVP) Nanoparticles Incorporated in Polymer Films and Their Optical Properties

**DOI:** 10.3390/ma6062119

**Published:** 2013-05-24

**Authors:** Toshio Itoh, Toshio Uchida, Noriya Izu, Ichiro Matsubara, Woosuck Shin

**Affiliations:** National Institute of Advanced Industrial Science and Technology (AIST), Shimo-shidami, Moriyama-ku, Nagoya 463-8560, Japan; E-Mails: to-uchida@aist.go.jp (T.U.); n-izu@aist.go.jp (N.I.); matsubara-i@aist.go.jp (I.M.); w.shin@aist.go.jp (W.S.)

**Keywords:** core–shell nanoparticles, cerium oxide, poly(vinylpyrrolidone), pentaerythritol triacrylate, UV-blocking film, haze

## Abstract

We fabricated hybrid films of pentaerythritol triacrylate (PETA) with core–shell ceria/poly(vinylpyrrolidone) (PVP) nanoparticles, which consist of cerium oxide as the core and PVP as the shell, and investigated the film optical properties. In this study, we used ceria/PVP nanoparticles with average diameters of 37, 49 and 91 nm. We obtained translucent films consisting of PETA with core–shell ceria/PVP nanoparticles. The core–shell ceria/PVP nanoparticles can reduce the transmittance of near-ultraviolet light. The transmittance of visible light and haze values depends not only on the thickness of the films, but also on the average diameter of the nanoparticles. A SEM observation and the optical analyses prove that the core–shell ceria/PVP nanoparticles do not aggregate into the PETA matrix.

## 1. Introduction

Transparent films are vital to enhance the coating, hard coat [1], UV-blocking [2] and anti-reflection properties [3,4,5,6,7] for optical devices. To improve the optical properties of the films, the addition of inorganic grains, as potential fillers, in organic polymer matrixes have been investigated [2,8,9,10].

**Figure 1:**
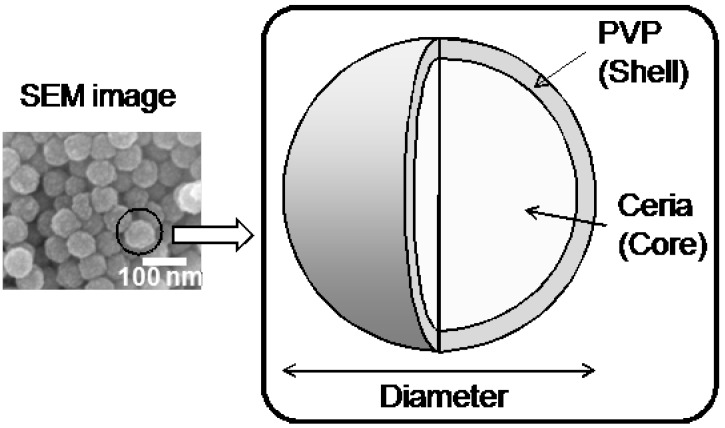
Depiction of microstructure of core–shell ceria/poly(vinylpyrrolidone) (PVP) nanoparticles.

Cerium oxide possesses specific optical properties, such as high ultraviolet absorbance [11] and high refractive index [12,13]. It has been reported that cerium oxide nanoparticles can be synthesized by thermal hydrolysis, [14] the polyol method [15,16,17,18], and the emulsion method [19]. Specifically, the diameter of ceria/polyvinylpyrrolidone (PVP) nanoparticles can be controlled by the molecular weight of PVP to obtain the nanoparticles with narrow size distributions [16,17]. The nanoparticles have core–shell type structures, in which the core and shell consist of cerium oxide and PVP, respectively (Figure 1; we have already reported their TEM image in [16]). The cerium oxide−core particles are formed from non-porous cerium oxide primary particles with diameters of 2–4 nm [18]. The dried nanoparticles powder is easily dispersed in water or alcohol without a dispersant because the PVP-shell has high affinity for polar solvents. The suspension of the core–shell ceria/PVP nanoparticles is stable for several weeks [16]. The PVP-shell is hardly soluble in the solvent because PVP on the surface of the cerium oxide–core forms cross-linked structures during the nanoparticle synthesis [20]. The rigid cross-linked structure of PVP is thought to ensure the stability of the suspension so that a uniform thin film of nanoparticles can be coated. The mechanical strength of the thin film with ceria/PVP nanoparticles without polymer matrix is relatively low and any polymeric curing matrixes can enhance the strength.

In the present study, we dispersed the core–shell ceria/PVP nanoparticles into the precursor of a transparent polymer matrix and prepared polymer films with the nanoparticles for the UV-blocking property of ceria. Since the nanoparticle has the PVP-shell, it is expected that the core–shell ceria/PVP nanoparticles also possess good affinity for polymer matrix. We carried out the preparation of inks which consist of pentaerythritol triacrylate (PETA), one of the hard coating materials [1], and the core–shell ceria/PVP nanoparticles. We finally investigated the effect of the nanoparticle diameter on the transmittance and haze values as well as the UV-blocking property of the hybrid films.

## 2. Results and Discussion

### 2.1. Effect of Affinity between Shell–PVP and Polymer Matrix

**Figure 2:**
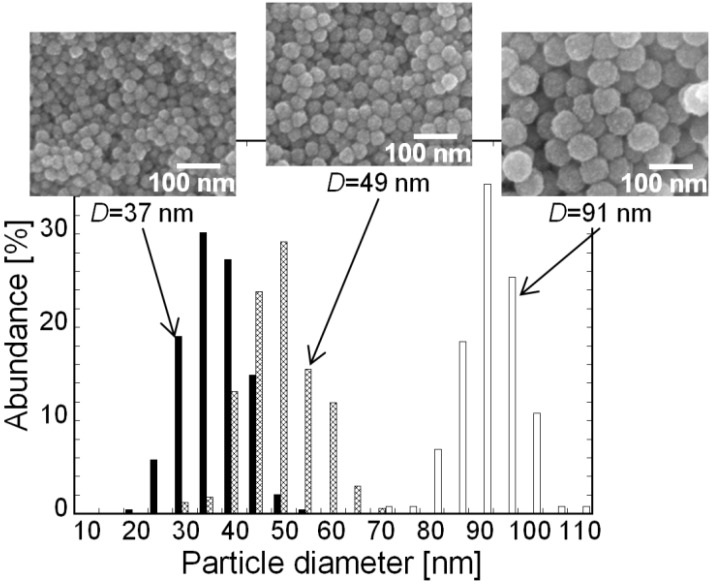
Particle size distribution of the nanoparticles from field-emission scanning electron microscopic (FE-SEM) observations. FE-SEM images and *D* of nanoparticles are also indicated on the panel.

The core–shell ceria/PVP nanoparticles were synthesized in accordance with our previous report [17]. The average diameter of the nanoparticles can be controlled by the average molecular weight of PVP during the synthesis of nanoparticles. The diameter was estimated from field-emission scanning electron microscopic (FE-SEM) images. We prepared nanoparticles having average diameters (*D*) of 37, 49 and 91 nm. Figure 2 shows the particle size distribution of the nanoparticles.

**Figure 3:**
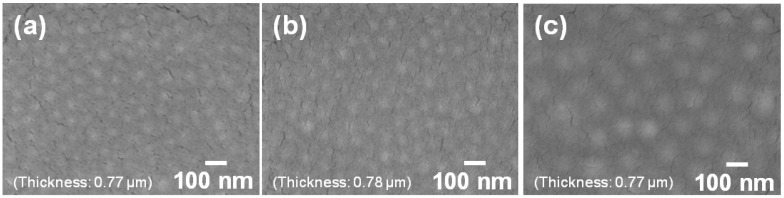
SEM images of the surfaces of the nanoparticles/pentaerythritol triacrylate (PETA) hybrid films on the PET substrates. Thickness of all the hybrid films is around 0.77 μm. *D* of containing nanoparticles are (**a**) 37 nm; (**b**) 49 nm; and (**c**) 91 nm.

**Figure 4:**
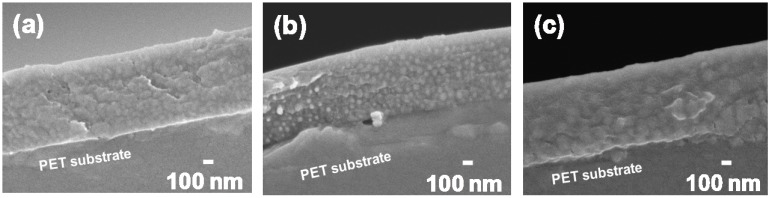
SEM images of the nanoparticles/PETA hybrid films on the PET substrates in cross section. Thickness of all the hybrid films is around 0.77 μm. *D* of containing nanoparticles are (**a**) 37 nm; (**b**) 49 nm; and (**c**) 91 nm.

Figure 3 shows the SEM images of the surface of the core–shell ceria/PVP nanoparticle/PETA hybrid films on a poly(ethylene terephthalate) (PET) substrates. The thickness of all the hybrid films is around 0.77 μm. In all hybrid films, the nanoparticles blend into the polymer films at high density, and no aggregation of the nanoparticles and the flat surfaces were observed. The same structures were observed in the cross sections shown in Figure 4. The nanoparticles dispersed uniformly into the PETA matrix in both lateral and vertical directions. During preparing the hybrid films, the core–shell ceria/PVP nanoparticles were dispersed in the methyl *i*-butyl ketone (MiBK)/3-methoxy-3-methylbutanol (MMB) mixture, and their dispersing properties were maintained even after mixing of the nanoparticle suspension and solution of PETA with 1-hydroxycyclohexyl phenyl ketone (HCPK), as a photopolymerization initiator. The hybrid films are almost translucent, excluding impurities or bubbles as shown in Figure 3 and Figure 4. The hybrid films were not blemished by the simple scratch test that involved rubbing of the film surface with a sharp tool. The hybrid films adhered closely to the PET substrate, though PETA was present within the nanoparticles. Sub-micro-cracks on the surface of the hybrid films are generated by electron radiation during the SEM observations, as shown in Figure 3.

**Figure 5:**
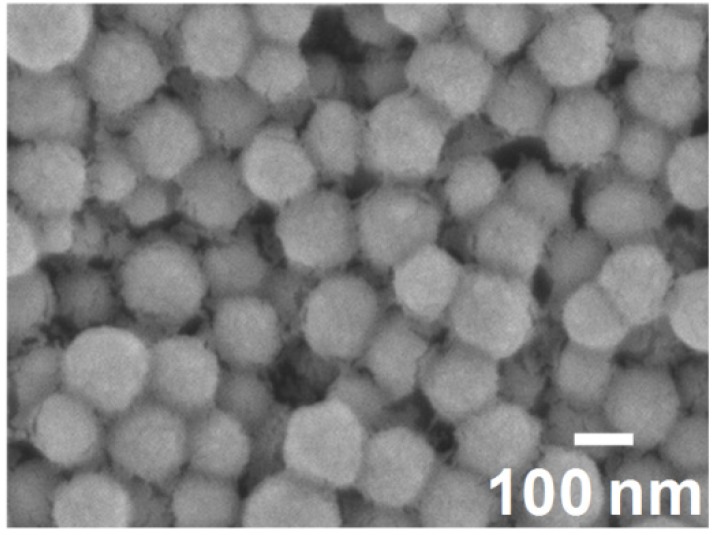
SEM images of the surfaces of the calcined nanoparticles/PETA hybrid films on the PET substrates.

Figure 5 shows the SEM images of the surface of the shell of PVP-reduced nanoparticle/PETA hybrid films on the PET substrates. Before preparing the nanoparticle suspension, the core–shell ceria/PVP nanoparticles were heated at 200 °C for 4 h in order to reduce the amount of PVP-shell. The purpose of using the shell-reduced nanoparticle is to increase the amount of ceria into the hybrid films. However, it was observed that the shell-reduced nanoparticles were hardly enwrapped by the PETA matrix, and the surfaces of the hybrid films were roughness.

From these results, it should be explained that the PVP as the shell of nanoparticles plays an important role of being enwrapped by the PETA matrix. The affinity of PVP to PETA can be explained by the theory of the solubility parameter. The solubility of the polymer into the solvent is improved when the solubility parameter values of the polymer and the solvent are close to each other [21]. The solubility parameter of MiBK has been reported to be 17.2 (MPa)^1/2^, which value is based on their heat of vaporization [21]. The solubility parameters of PVP, PETA, and MMB are given by Equation (1),

(1) $$\delta =\frac{\rho }{M}\times \sum F_{i}$$

where *δ*, *ρ,* *M*, and *F_i_* denote the solubility parameter, density, molecular weight of unit, and group molar attraction constants, respectively [21,22]. The solubility parameters of PVP, PETA, and MMB are calculated to be 17.3, 16.2, and 16.7 (MPa)^1/2^, respectively, using *F_i_* constants reported from Small and Hoy [21,22,23]. These values can explain that PVP possesses good affinity to PETA as well as MiBK/MMB solvent mixture. However, the shell-reduced nanoparticles were hardly enwrapped by the PETA matrix. The PVP of the shell-reduced nanoparticles would be denatured and the solubility parameter of the surface PVP would vary in response to heating. In this study we did not successfully increase the amount of ceria in the hybrid films, so the synthesis of ceria nanoparticles with thin PVP-shell will be investigated in the future.

### 2.2. Optical Properties of the Hybrid Films

We have developed a method for the preparation of a hard coated polymer film including core–shell ceria/PVP nanoparticles. Subsequently, we investigated the optical properties of the hybrid films.

**Figure 6:**
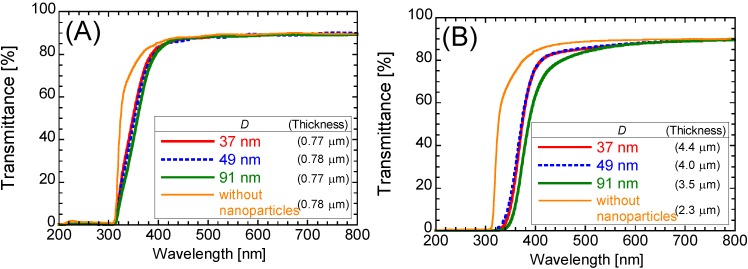
UV-Vis transmittance spectra of the nanoparticles/PETA hybrid films on the PET substrates. Film thicknesses: (**A**) around 0.77 μm; (**B**) around 4.0 μm.

Figure 6 compares the UV-Vis. transmittance spectra of the nanoparticles/PETA hybrid films with two different film thicknesses on the PET substrates. For comparison, the spectrum of the 0.77 μm thick PETA film without the nanoparticles is also shown in Figure 6. In Figure 6A, the thickness of all films is around 0.77 μm. Approximately 88% of visible light can pass through the PET substrate and PETA film with and without nanoparticles. The nanoparticles of the hybrid films reduced the transmittance of near-ultraviolet light between 320 and 380 nm wavelengths. The thick hybrid films having around 4 μm of film thickness possess an excellent UV-blocking property, as shown in Figure 6B. The spectrum of the hybrid film including nanoparticles with *D* = 91 nm is lower than those including nanoparticles with *D* = 49 and 37 nm. As the film thickness increased, noticeable reduced transmittance of light over 380 nm wavelengths was observed. Specifically nanoparticles with *D* = 91 nm, the hybrid film has a yellowish color because of increasing film thickness. The hybrid films including nanoparticles with *D* = 49 and 37 nm are transparent and colorless.

**Figure 7:**
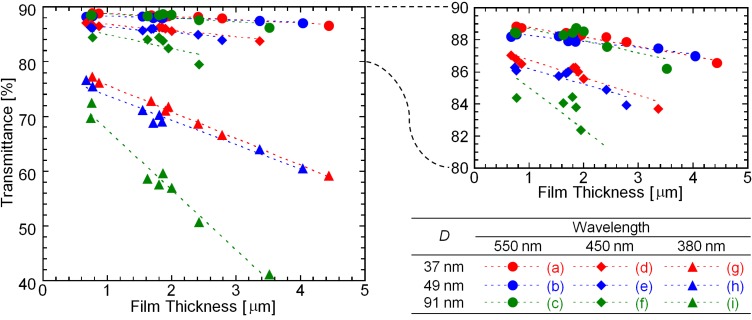
Transmittance of lights with (**a**–**c**) 550 nm; (**d**–**f**) 450 nm; and (**g**–**i**) 380 nm wavelengths through the nanoparticle/PETA hybrid films and PET substrates. The *D* of the nanoparticles are (**a**,**d**,**g**) 37; (**b**,**e**,**h**) 49; and (**c**,**f**,**i**) 91 nm.

Figure 7 shows the transmittance of lights with 380, 450, and 550 nm wavelengths through the nanoparticle/PETA hybrid films and PET substrates. The transmittance of green light with a 550 nm wavelength is relatively independent of the thickness of the hybrid films. In the case of the bluish violet light with a 450 nm wavelength, its transmittance is also approximately independent of the thickness of the hybrid films containing nanoparticles with *D* = 37 and 49 nm. However, the thickness of the hybrid film containing nanoparticles with *D* = 91 nm affects the transmittance intensity of the bluish violet light. The transmittance intensity of the light bordering between violet and ultraviolet lights with a 380 nm wavelength can be reduced by thickening the polymer film using not only nanoparticles with *D* = 91 nm, but also using nanoparticles with *D* = 37 and 49 nm.

**Figure 8:**
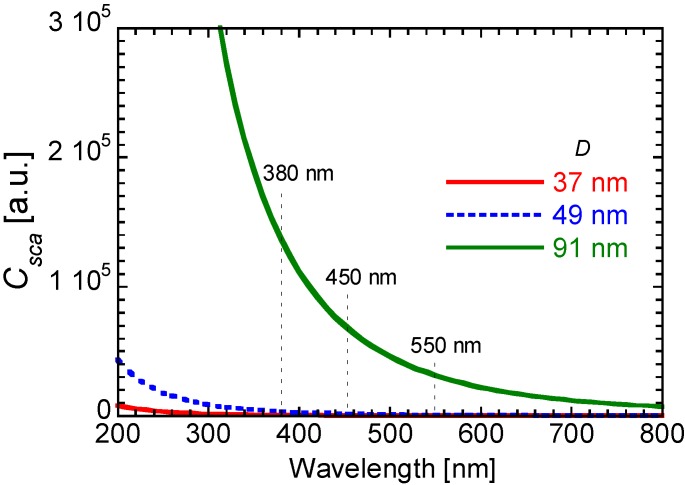
Model of scattering coefficient of the nanoparticle/PETA hybrid films using Equation (2).

These results are explained by Rayleigh scattering theory. Scattering coefficient, *C_sca_*, is defined as the following Equation (2),

(2) Csca=83(2πnmD2λ)4⋅((np/nm)2−1(np/nm)2+2)2⋅π(D2)2

where *n_m_*, *n_p_*, and *λ* are refractive index of polymer matrix, refractive index of particle, and wavelength, respectively. Figure 8 is a model of scattering coefficient using Equation (2). In cases of 380, 450, and 550 nm wavelength, Figure 8 shows that the scattering coefficient values from the *D* = 91 nm hybrid film are extremely larger than those from the *D* = 37 and 49 nm hybrid films. Therefore, the transmittance intensity should be strongly affected by the light scattering effect.

**Figure 9:**
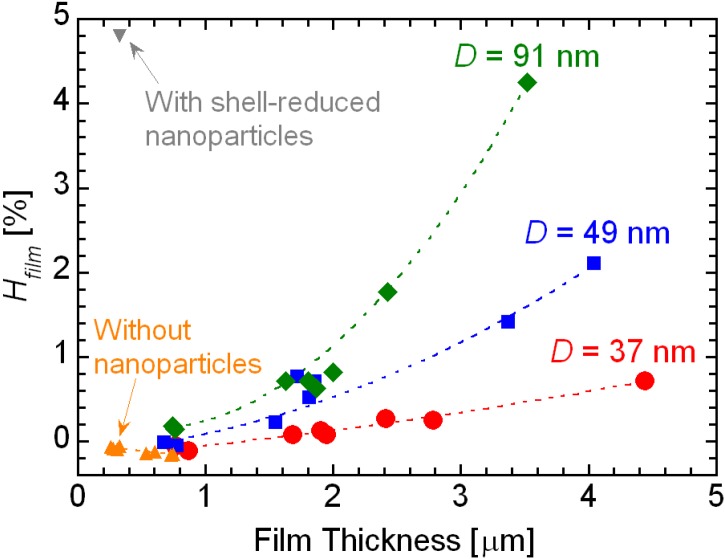
Haze values (*H*_film_) of the nanoparticles/PETA hybrid films and the PETA film without nanoparticles on the PET substrates.

Figure 9 shows the haze values, *H*_film_, of the nanoparticles/PETA hybrid films on the PET substrates. The haze value of the film, *H*_film_, is defined as the following Equation (3),

*H*_film_ = *H*_sample_ − *H*_PET_ (3)

where *H*_sample_ and *H*_PET_ are the haze values of the film with the PET substrate and the PET substrate without the film, respectively. The haze value of the hybrid film including the shell-reduced nanoparticles tends to be larger than the hybrid films including the original nanoparticles because of rough surface. In the case of the PETA films without the nanoparticles, the haze values are negative, as shown in Figure 8, indicating that light scattering from fine scratches and fine rough surface of PET substrate would be suppressed by the PETA-coating. In addition, hybrid films around 0.8 μm in thickness including those with nanoparticles having *D* = 37 and 49 nm possesses negative haze values. It is, therefore, explained that the nanoparticles hardly affect the flatness of the films. The SEM observations in Figure 3 and Figure 4 also show that the surface of the hybrid films is smooth and flat. The surface conditions of the hybrid films are independent of the haze values. Therefore, the haze values of the hybrid films depend on the diameter of the nanoparticles. When the hybrid films including nanoparticles with *D* = 91 nm become thick, the haze values of the films become drastically high. We cannot see a difference in transmittance between hybrid films with nanoparticles between *D* = 37 and 49 μm. However, the difference is clearly observed between films consisting of nanoparticles with *D* = 37 and 49 μm by the haze value analysis.

It is generally known that the diameter of nanoparticles in a polymer matrix should be approximately less than 1/10 of the wavelength of the light to avoid Rayleigh scattering [24]. Transmittances of short wavelength visible lights and haze values decrease when large nanoparticles are included in the polymer matrix. In the study of the optical properties, the transmittance of lights and the haze values of the hybrid films strongly depend on the grain size of the including nanoparticles, which is good agreement with general light scattering theory, e.g*.*, Rayleigh scattering. That is, these optical analyses also prove the nanoparticles do not aggregate in the PETA matrix.

## 3. Experimental Section

### 3.1. Synthesis of Core–Shell Ceria/PVP Nanoparticles/PETA Hybrid Films

**Figure 10:**
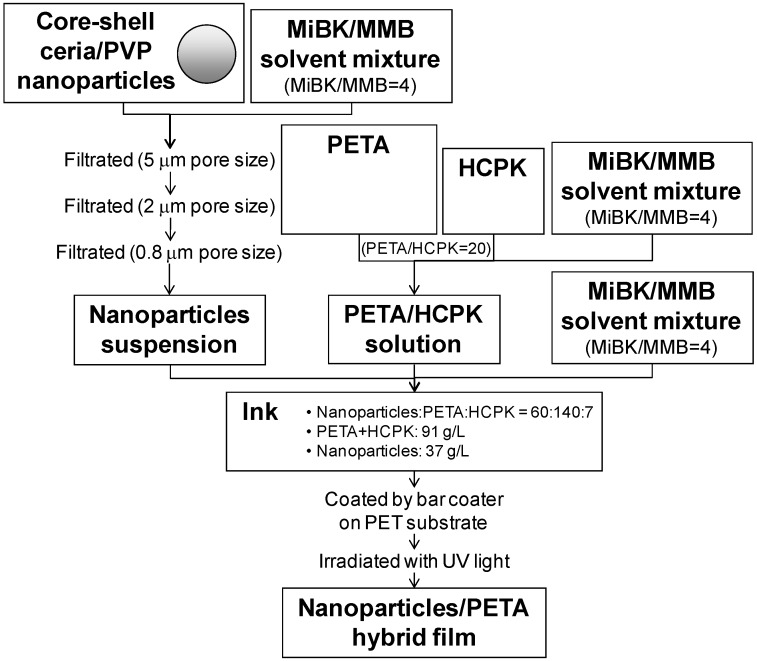
Preparation scheme for the nanoparticles/PETA hybrid films.

The core–shell ceria/PVP nanoparticles were synthesized in accordance with our previous report [17]. In the case of the shell-reduced nanoparticles, around 100 nm of average grain size of nanoparticles were heated at 200 °C for 4 h. The reducing amount of PVP was around 40%, which was confirmed by thermogravimetric analysis. Figure 10 shows a preparation scheme for the nanoparticles/PETA hybrid films. The obtained nanoparticles were dispersed in a solvent mixture of MiBK (Wako Pure Chemicals) and MMB (Kuraray). The volume ratio of MiBK/MMB was 4. The suspensions were filtered three times through filters having 5, 2, and 0.8 μm pore sizes. PETA as a precursor of the polymer film (57% of triester; Shin-Nakamura Chemical) and HCPK as a photopolymerization initiator (Chiba Speciality Chemicals) were dissolved in another solvent mixture of MiBK/MMB. The weight ratio of PETA/HCPK was 20. Inks of films were prepared to mix the nanoparticles suspension, the PETA/HCPK solution, and the MiBK/MMB solvent mixture. The weight ratio of nanoparticles:PETA:HCPK was 60:140:7, and the concentrations of the solutes (PETA and HCPK) and nanoparticles were 91 and 37 g/L, respectively. Therefore, the concentration of nanoparticles was 29.0 wt % in the hybrid film when the solvent was evaporated completely. In a similar manner, inks without the nanoparticles were also prepared. The inks were coated on the PET substrate (Toray), whose thickness is 0.1 mm, using a bar coater RK Print Coat Instrument K101. The thickness of the films was controlled by the volume of the inks and the wire diameter of the bars. After being coated, the substrates were irradiated for 8 min with UV light using a 1.5 kW mercury lamp equipped with an IR cut filter for the polymerization of PETA.

### 3.2. Analysis

The surface and cross section of the hybrid films were observed by FE-SEM, using a JEOL JSM-6335FM microscope. The thickness of the hybrid film was estimated from the FE-SEM images. Transmittance of light by the hybrid film on the PET substrate was measured using a JASCO V-670 UV-Vis. spectrophotometer. Meanwhile, the haze value was measured using a Nippon Denshoku Industries NDH 5000 haze meter, equipped with a white light emitting diode as the light source.

## 4. Conclusions

We performed a study to prepare novel hybrid films, consisting of PETA and core–shell ceria/PVP nanoparticles. The SEM images showed that the nanoparticles can be dispersed uniformly with a high density and without aggregation into the PETA matrix. The nanoparticles into the films reduce the transmittance of near-ultraviolet light. The UV-Vis. transmittance spectra and haze analysis show that the transmittance property of hybrid films depends on not only the thickness of the hybrid films but also the diameter of the nanoparticles, which is good agreement with general light scattering theory. The SEM images and the optical analyses prove non-aggregation of the nanoparticles into the PETA matrix. The PETA film with the core–shell ceria/PVP nanoparticles shows excellent UV-blocking and visible light-transmitting properties.
